# Nitropyridines in the Synthesis of Bioactive Molecules

**DOI:** 10.3390/ph18050692

**Published:** 2025-05-07

**Authors:** Alexey Starosotnikov, Maxim Bastrakov

**Affiliations:** N.D. Zelinsky Institute of Organic Chemistry, Russian Academy of Sciences, Leninsky Prosp. 47, 19991 Moscow, Russia; maxim82@ioc.ac.ru

**Keywords:** nitropyridines, biological activity, heterocycles, nitro group, coordination compounds, radiolabeled compounds

## Abstract

Pyridines are one of the most important and promising classes of N-heterocycles actively studied in modern organic and medicinal chemistry; in particular, pyridine is a privileged structural motif in drug design. From a synthetic organic chemistry perspective, nitropyridines can be considered as convenient and readily available precursors for a wide range of mono- and polynuclear heterocyclic systems demonstrating diverse activities, such as antitumor, antiviral, anti-neurodegenerative, etc. This review is an analysis of the literature on the use of nitropyridines for the synthesis of biologically active compounds, covering the period from 2015 to the present.

## 1. Introduction

Pyridine-based ring systems represent a promising class of nitrogen heterocycles, which are actively studied in both medicinal and agricultural chemistry. In particular, pyridine serves as a privileged structural motif in drug design. As of 2021, approximately 14% of *N*-heterocyclic drugs approved by the FDA contain a pyridine moiety. Notable examples include isoniazid, omeprazole, enpiroline, piroxicam, tacrine, and many others [1]. Beyond their application in pharmacology [2], pyridine derivatives are in demand in material science [3,4]; they are used as dyes [5,6,7], in catalysis [8,9], and in other fields.

Among numerous pyridine derivatives, nitropyridines deserve particular attention. Over the past few decades, nitropyridines have been shown to exhibit diverse biological activities, such as the inhibition of cytosolic thioredoxin reductase 1, which serves as a mechanistic basis for anticancer therapy [10]. They also inhibit the human platelet Na^+^/H^+^ exchanger [11] and display antifungal activity [12]. Furthermore, nitropyridines have been utilized as precursors in the synthesis of various compounds, including azaindole hydroxamic acids, which are potent HIV-1 integrase inhibitors [13]; azaphenothiazines, which act as potent CNS-depressants [14]; and ligands for α-synuclein [15]. Remarkably, 3,5-dinitropyridine has been identified as a metabolite of *Lactobacillus kunkeei* [16].

Despite the availability and wide synthetic potential of nitropyridines, this class of compounds has not received much attention in the review literature. There is a review devoted to the synthesis of 3-nitropyridines from acyclic synthones and through the transformation of other heterocyclic rings, published as early as in 1991 [17]. Additionally, the synthesis and reactions of dinitropyridines were reviewed recently [18].

This review encompasses recent examples of the synthesis of bioactive nitropyridines, as well as their use as intermediates in the synthesis of promising biologically active compounds. For convenience, the considered methods for the preparation of target compounds are organized into separate subsections: the syntheses of nitropyridine-containing bioactive molecules, the formation of metal complexes with nitropyridine ligands, reactions involving nitro group(s) (where nitropyridines serve as intermediates), and the synthesis of radiolabeled compounds for the monitoring and treatment of pathological conditions.

## 2. Synthesis of Bioactive Nitropyridines

Many drugs have been created on the basis of aromatic nitro compounds [19]. In this section, syntheses of nitropyridine-containing bioactive molecules are considered.

**Scheme 1:**
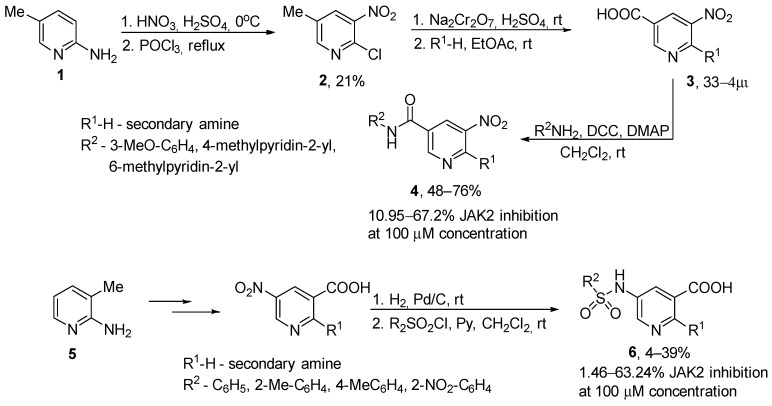
Synthesis of potent JAK2 inhibitors **4** and **6**. Data from [20].

A number of potent Janus kinase 2 (JAK2, a non-receptor tyrosine kinase) inhibitors were synthesized by Cho et al. [20]. 2-Chloro-5-methyl-3-nitropyridine **2**, obtained from 2-amino-5-methylpyridine, was oxidized to carboxylic acid, followed by nucleophilic substitution of the activated chlorine atom with secondary amines (Scheme 1). The resulting carboxylic acids **3** were coupled with aromatic amines in the presence of *N*,*N*′-dicyclohexylcarbodiimide (DCC), thus affording target compounds **4** in moderate yields. Similarly, starting with isomeric 2-amino-3-methylpyridine **5**, a series of sulfamides **6** were synthesized. The most potent compounds inhibited JAK2 with IC_50_ 8.5–12.2 µM.

**Scheme 2:**
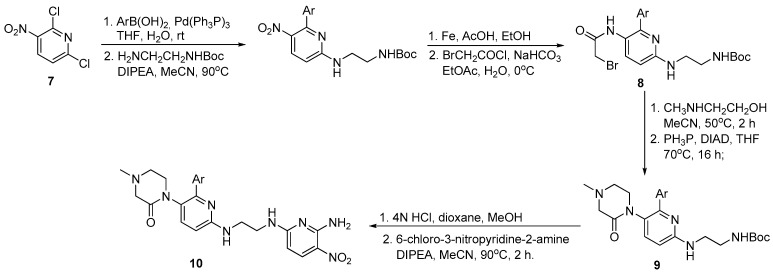
Bussiere’s method for the synthesis of GSK3 inhibitors **10** [21].

Another application of nitropyridine derivatives was demonstrated by Bussiere et al. [21]. The authors synthesized a novel series of heterocyclic compounds that have been shown to inhibit glycogen synthase kinase-3 (GSK3), which mediates the addition of phosphate molecules onto serine and threonine amino acid residues. In 2,6-dichloro-3-nitropyridine **7**, both chlorine atoms were successively substituted with aryl (Suzuki coupling) and aminoethylamine fragments (Scheme 2). Then, the nitro group was reduced to amine, which in turn was acylated with bromoacetyl chloride to give the corresponding amide **8**. Further alkylation and intramolecular Mitsunobu reaction afforded intermediate **9**, which, after Boc protection cleavage and reaction with 2-amino-6-chloro-3-nitropyridine, resulted in target compounds **10**. The most active GSK3 inhibitor from this series contains 2,4-dichlorophenyl moiety (Ar = 2,4-Cl_2_-C_6_H_3_); IC_50_ 8 nM, EC_50_ 0.13 μM.

**Scheme 3:**
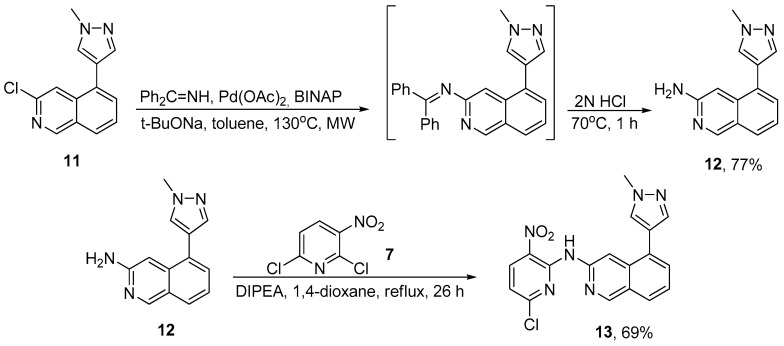
Synthesis of potent kinase p70S6Kβ inhibitor **13**. Adopted from [22].

2,6-Dichloro-3-nitropyridine **7** also served as a key intermediate in the synthesis of a potent kinase p70S6Kβ inhibitor [22]. The Buchwald–Hartwig arylamination of 3-chloroisoquinoline derivative **11** with benzophenonimine, followed by hydrolysis of the imine, produced 3-aminoisoquinoline intermediate **12**, which was then reacted with 2,6-dichloro-3-nitropyridine **7** to yield target compound **13** as a product of the regioselective nucleophilic substitution of chlorine in position 2 of the pyridine nucleus (Scheme 3). Compound **13** did not demonstrate the expected inhibitory activity against kinase MPS1; however, it inhibited the kinase p70S6Kβ with IC_50_ 444 nM. The authors considered this compound as a starting point for the development of p70S6Kβ inhibitors.

**Scheme 4:**
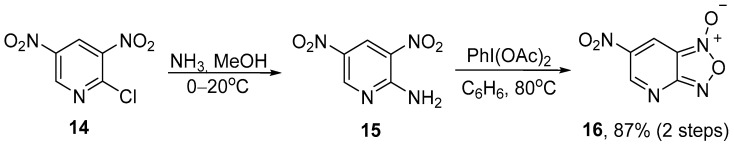
A method for the synthesis of 4-aza-6-nitrobenzofuroxan **16** [24].

3-Nitropyridine fused with a furoxan ring (4-aza-6-nitrobenzofuroxan **16**) was studied as a potential dual-action HIV-1 inhibitor [23]. Compound **16** inhibited two HIV-1-enzymes: integrase and the RNase H domain within reverse transcriptase. In particular, its activity levels (IC_50_) against both catalytic activities of integrase (strand transfer reaction and 3′ processing) were 190 ± 30 μM and 60 ± 15 μM, respectively, while an IC_50_ of 90 ± 20 μM was found for RNase H inhibition. This compound was synthesized by a two-step method, starting from 2-chloro-3,5-dinitropyridine **14** [24] (Scheme 4).

**Scheme 5:**
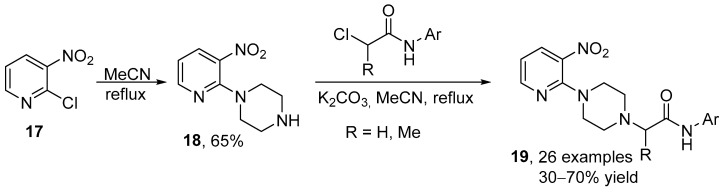
Synthesis of nitropyridine-containing potential urease inhibitors **19**. Data from [25].

3-Nitropyridylpiperazine derivatives were synthesized and evaluated as potential urease inhibitors for the treatment of gastric diseases [25]. Synthesis was accomplished by the reaction of 2-chloro-3-nitropyridine **17** with piperazine and further N-alkylation with various aryl 2-chloroacetamides or 2-chloropropioamides (Scheme 5). The IC_50_ values of the most promising compounds **19** are about 2.0–2.3 μM against jack bean urease, which is much lower than the IC_50_ of thiourea (23.2 μM), a standard urease inhibitor.

**Scheme 6:**
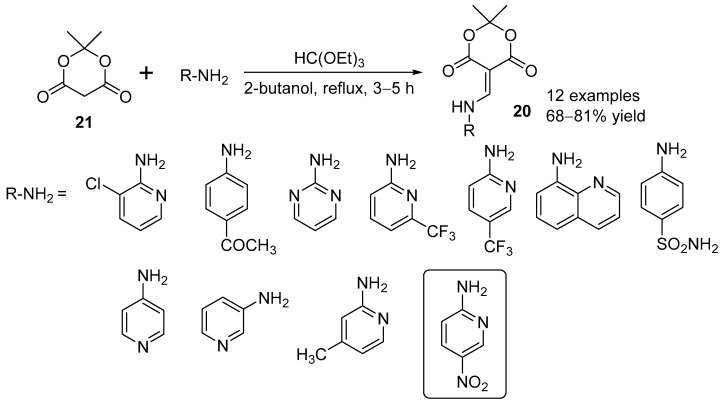
Synthesis of novel urease inhibitors **20**. Data from [26].

New 5-aryl(hetaryl)-2,2-dimethyl-1,3-dioxane-4,6-dione derivatives **20** were was newly synthesized by the condensation of Meldrum’s acid (**21**), triethyl orthoformate, and substituted aromatic or heteroaromatic amines, and then evaluated for chymotrypsin and urease inhibition [26] (Scheme 6). Among the synthesized compounds, the 5-nitropyridin-2-yl derivative exhibited dual inhibition activities, with an IC_50_ value of 8.67 ± 0.1 μM against chymotrypsin and 29.21 ± 0.98 μM against urease.

In addition, (5-nitropyridin-2-yl)imine 1,3,4-thiadiazole hybrids were designed as potent and selective factor IXa inhibitors and proven to be prospective candidates in the search for anticoagulant drugs [27]. These compounds have higher inhibition levels and lower IC_50_ values with respect to similar compounds with no nitro group in the pyridine cycle.

The crystal structures of 4-phenylsulfanylbutyric acid and its cocrystal with 2-amino-5-nitropyridine have been studied [28]. The in vitro DNA cleaving and antimicrobial activity of the cocrystal against a number of Gram-positive (*S. aureus*, *S. pneumoniae*) and Gram-negative bacteria (*E. coli*, *P. aeruginosa*, *P. vulgaris*) were higher than those of 4-phenylsulfanylbutyric acid alone.

Silver nanoparticles (AgNPs) are recognized as effective agents against biofilms formed by *P. aeruginosa*. However, when combined with 4-nitropyridine-N-oxide (4-NPO), they demonstrate even more significant antibiofilm efficiency compared to AgNPs alone [29]. The primary advantage of the AgNP-4-NPO combination as an antimicrobial agent lies in its multi-level effects on bacterial physiological processes. This synergetic approach interferes with the quorum sensing system and inhibits the secretion of extracellular polymeric substances, which are crucial for *P. aeruginosa* to resist biofilm formation.

The effect of irreversible biofouling of the reverse osmosis membrane and the inhibitory effect of biofilm inhibitors on irreversible biofouling were investigated using *P. aeruginosa* as model bacteria [30]. The biofilm inhibitor 4-NPO, at concentrations of 30–50 μM, significantly inhibited the biofilm formation and reduced the irreversible biofouling resistance without affecting microbial growth in reverse osmosis processes.

The influence of bacterial quorum-sensing inhibitors on the formation of diatom biofilm has been studied [31]. 4-Nitropyridine-N-oxide demonstrated an inhibitory effect on the biofilm formation of *Cylindrotheca* sp. In addition, it effectively decreased the risk of human bacterial pathogens in soil [32].

**Scheme 7:**
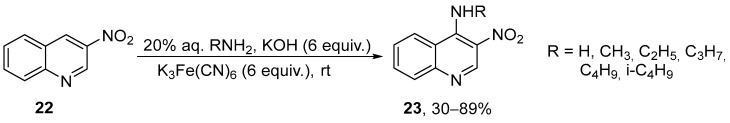
S_N_^H^ alkylamination of 3-nitroquinoline **22**. Data from [33].

The synthesis of antibacterial compounds based on nitropyridines was described very recently [33]. The authors studied the oxidative S_N_^H^ alkylamination of nitroquinolines and nitroisoquinolines in an aqueous medium and found the process to be selective, depending solely on the position of the nitro group. For example, 3-nitroquinoline **22** was aminated in position 4 by the reactions with primary amines and K_3_Fe(CN)_6_ at room temperature (Scheme 7). However, the most active compounds were obtained from 5- and 8-nitroquinolines.

**Scheme 8:**
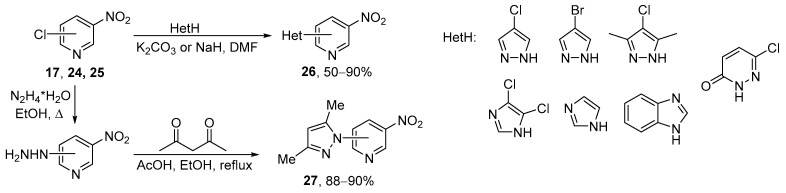
Synthesis of antibacterial 3-nitropyridines [34].

Novel 3(5)-nitropyridines, functionalized with azole or pyridazine moieties, were synthesized from 2-, 4-, and 6-chloro-3-nitropyridines (**17**, **24**, and **25**) [34] (Scheme 8). The authors observed moderate antibacterial activity of pyrazole and imidazole derivatives against *S. aureus* and *E. coli*, as well as an antiprotozoal effect against *Colpoda steinii*.

**Scheme 9:**
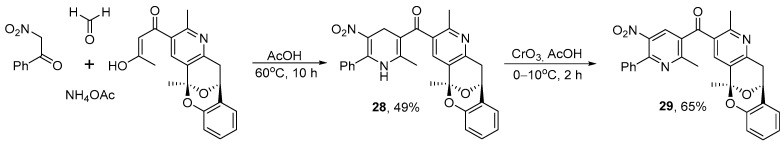
Preparation of epoxybenzooxocino[4,3-*b*]pyridine derivatives. Data from [35].

Kulakov and coworkers reported on the multicomponent synthesis and evaluation of the bacteriostatic properties of epoxybenzooxocino[4,3-*b*]pyridine derivatives [35]. Hantsch 1,4-dihydropyridine synthesis was carried out using 2-nitroacetophenone, paraformaldehyde, ammonium acetate, and substituted β-dicarbonyl compound as reactants (Scheme 9). The resulting compound **28** was oxidized with a solution of CrO_3_ in acetic acid to yield 5-nitropyridine **29**. Both compounds were active against *Mycobacterium bovis* 14, with MIC values of 12.5–50 μg/mL.

**Scheme 10:**
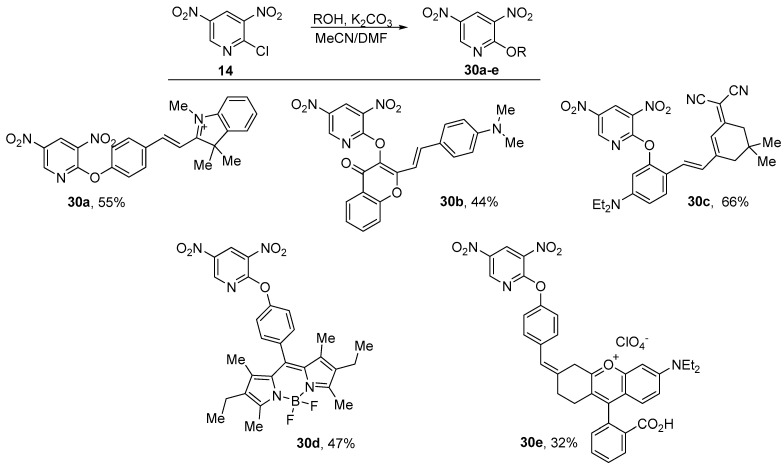
Synthesis of 3,5-dinitropyrydine containing fluorophores [36,37,38].

A number of articles have been published on the introduction of electron-deficient 3,5-dinitropyridine moiety into a fluorophore molecule (Scheme 10). The authors revealed an excellent electron sink of such compounds that allows for using it as novel fluorescent probes for the detection of biothiols [36,37,38]. Such molecules were prepared via nucleophilic substitution of chlorine in 2-chloro-3,5-dinitropyridine **14** under mild conditions.

**Scheme 11:**
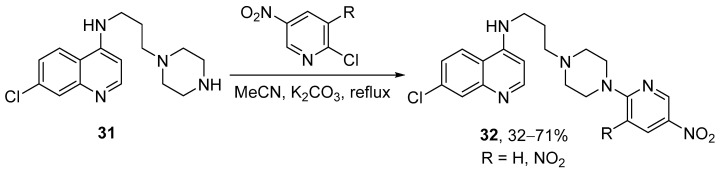
Synthesis of nitropyridine-based compounds with antimalarial activity. Data from [39].

Hybrid molecules containing chloroquine fragments linked with nitropyridyl fragments were reported to possess good antimalarial activity [39]. The target compounds **32** could be obtained by reacting chloroquine derivative **31** with the corresponding 3-R-2-chloro-5-nitropyridine (Scheme 11). The reactions proceeded in the presence of K_2_CO_3_ under reflux in MeCN. Some compounds demonstrated IC_50_ values below 5 nM.

**Scheme 12:**
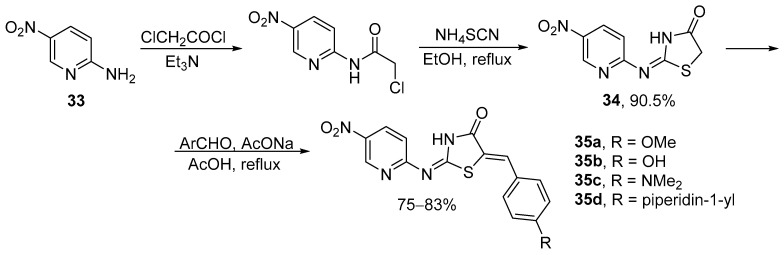
Synthesis of nitropyridine-linked 4-arylidenethiazolidin-4-ones **35**. Data from [40].

Nitropyridine linked 4-arylidenethiazolidin-4-ones, as potent anticancer agents, were synthesized from 2-amino-5-nitropyridine **33** [40]. It was reacted with chloroacetyl chloride, and then treated with ammonium thiocyanate to produce 5-nitropyridyliminothiazolidin-4-one **34**, which was reacted with aromatic aldehyde to yield the corresponding arylidene derivatives **35** in 75–83% yields (Scheme 12). The target compounds possessed high selectivity against certain cancer types: **35a** (R = OMe) was active against MCF-7 cells, with an IC_50_ of 6.41 μM, while piperidine derivative **35d** was active against HepG2 cells, with an IC_50_ of 7.63 μM.

**Scheme 13:**
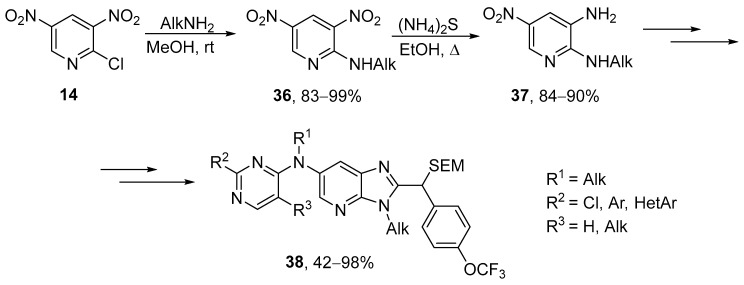
Synthesis of novel imidazo[4,5-*b*]pyridines [41].

Novel imidazopyridines as inhibitors of necroptosis were described in patent [41]. Commercially available 2-chloro-3,5-dinitropyridine **14** was used for the synthesis of the target compounds (Scheme 13). Nucleophilic substitution of the chlorine atom with the corresponding amine afforded compounds **36** in high yields. Selective reduction of the 3-NO_2_ group under the action of ammonium sulfide yielded compounds **37**. Further transformation led to the desired compounds **38**, which showed activity against TSQ-induced necroptosis at nanomolar concentrations.

**Scheme 14:**
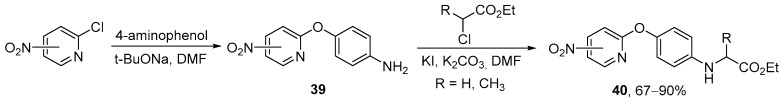
Synthesis of nitropyridine-containing herbicides. Data from [42].

The synthesis and herbicidal activity of novel nitropyridine-containing phenylaminoacetates and propionates were studied by Li and coworkers [42]. The chlorine atom in 2-chloro-3(5)-nitropyridines was substituted with 4-aminophenol, and the resulting aniline **39** was reacted with ethyl 2-chloroacetate or ethyl 2-chloropropionate to yield alkylation products **40** in moderate to high yields (Scheme 14). One of the synthesized compounds, namely ethyl 2-(4-(5-nitropyridin-2-yloxy)-phenylamino)propanoate, exhibited a high level of herbicidal activity on barnyard grass (IC_50_ 27.7 mg/L).

**Scheme 15:**
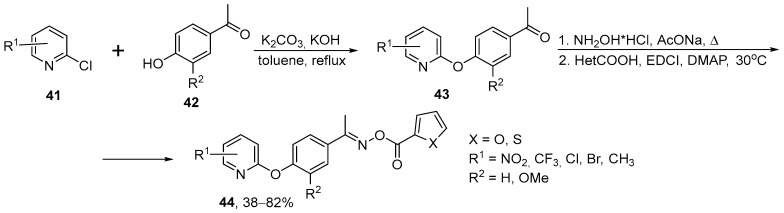
Synthesis of pyridyloxy-substituted acetophenone oxime ethers **44**. Data from [43].

Ye and coworkers synthesized and studied the herbicidal activity of some pyridyloxy-substituted acetophenone oxime ethers [43]. The reactions of 2-chloropyridines **41** with 4-hydroxyacetophenones **42** produced the corresponding 4-pyridyloxyacetophenones **43**, which were further converted into oximes and finally coupled with furan- or thiophene-2-carboxylic acids (Scheme 15). The resulting compounds **44** were tested for protoporphyrinogen oxidase inhibitory activity, since this is an important target for the discovery of novel herbicides. The nitropyridine derivatives demonstrated moderate activity (IC_50_ 3.11–4.18 μM).

**Scheme 16:**
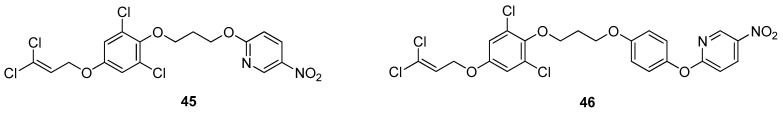
Nitropyridine insecticides [44].

2-Chloro-5-nitropyridine was used as the starting material in the synthesis of a new series of insecticides [44]. The method involves the nucleophilic substitution of a chlorine atom under the action of appropriate hydroxyl compounds. The two most promising derivatives, **45** and **46**, active against *M. separate*, *P. xylostella*, and *P. litura* with a median lethal concentration (LD_50_) of 4–12 mg/L, are presented below (Scheme 16).

## 3. Nitropyridines as Ligands in Bioactive Coordination Compounds

**Scheme 17:**
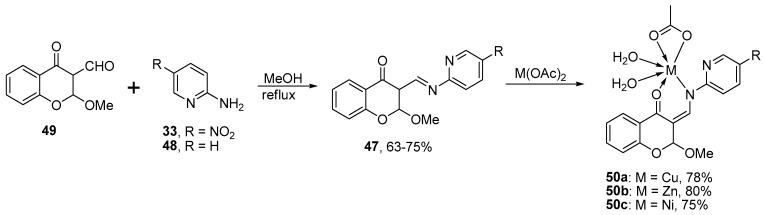
Synthesis of pyridine-chromene ligand **47** and its metal complexes. Data from [45].

Mohanan and coworkers reported on the synthesis and biological evaluation of novel mononuclear Cu(II), Zn(II), and Ni(II) complexes with 2-methoxy-4-chromanones as ligands [45]. These bidentate ligands (compounds **47**) were prepared from 2-amino-5-nitropyridine **33** or 2-aminopyridine **48** and 3-formyl chromene **49** (Scheme 17). The antifungal, antibacterial, antidiabetic, and DNA-binding abilities of the ligands and all complexes were tested. It was found that the nitropyridine-containing complexes **50a**–**c** possessed antimicrobial activity against *S. aureus*, *B. subtilis*, *P. aeruginosa*, and *E. coli*, which is comparable to the activities of ciproflaxin (mean zone diameters of 9.1–17.9 mm and 22–26 mm, respectively) and Nystatin against *C. albicans* (21.9–25.3 and 19 mm, respectively).

**Scheme 18:**
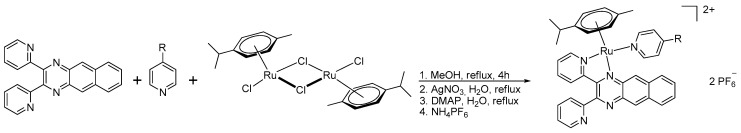
Synthesis of pyridine-chromene ligand **47** and its metal complexes [46].

The synthesis of Ru(arene) complexes, [(η6-p-cymene)Ru(dpb)(py-R)]^2+^ (dpb = 2,3-bis(2-pyridyl)benzoquinoxaline, py-R = 4-substituted pyridine, R = N(CH_3_)_2_, NH_2_, OCH_3_, H, COOCH_3_, and NO_2_) was also reported [46] (Scheme 18). The yields were not given. The complex with the 4-nitropyridine ligand demonstrated the highest DNA covalent binding capability under aerobic and anaerobic conditions, as well as moderate photocytotoxicity.

**Scheme 19:**
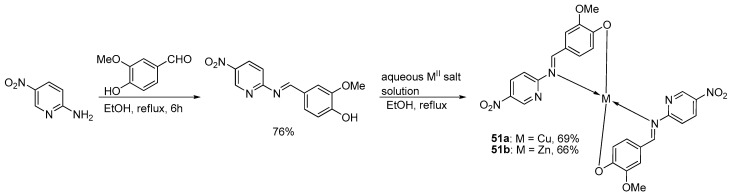
Synthesis of copper and zinc complexes **51** with (5-nitropyridin-2-yl)imine ligands. Adopted from [47].

The synthesis and biological activity of Cu(II) and Zn(II) complexes **51**, featuring (5-nitropyridin-2-yl)imine ligands, were investigated [47] (Scheme 19). The ability of both the complexes and the ligand to scavenge free radicals was evaluated; the Cu(II) complex exhibited a high level of antioxidant activity. Additionally, the ligand and its complexes were shown to effectively inhibit the α-glucosidase enzyme. Among them, the Cu(II) complex demonstrated the highest inhibitory activity, with an IC_50_ of 108 μg/mL), while the ligand itself had the lowest inhibitory activity, with an IC_50_ of 2.14 μg/mL.

**Scheme 20:**
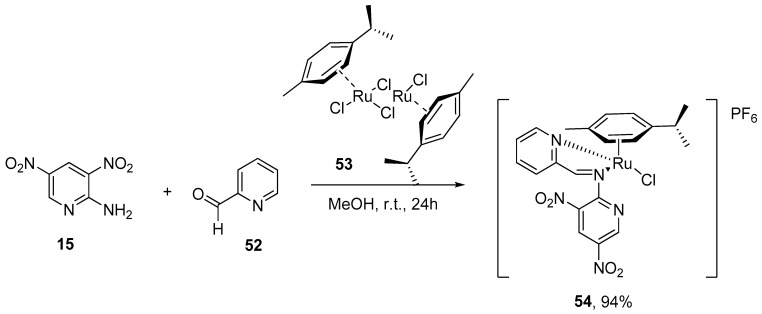
Synthesis of the Ru-arene-dinitropyridinylmethylene complex. Data from [48].

In 2016, Jadhav et al. reported a method for the synthesis of a ruthenium complex based on the 3,5-dinitropyridine moiety [48]. It can be prepared by the reaction of 2-amino-3,5-dintropyridine **15**, pyridine-2-carboxaldehyde **52**, and ruthenium(II) dichloro-*p*-cymene dimer **53** (Scheme 20). Stirring in methanol for 24 h resulted in complex **54** in 94% yield. Compound **54** possessed significant activity against breast cancer (MCF7) and the cervical epithelioid carcinoma cell line (HeLa).

**Scheme 21:**
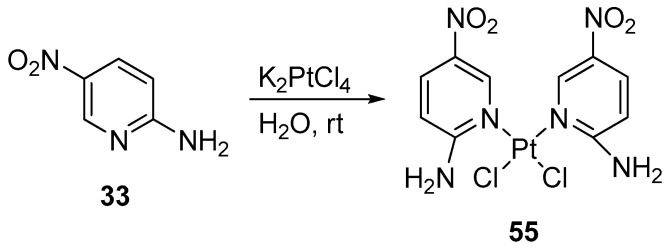
Preparations of Pt(II) complex of 2-amino-5-nitropyridine **33**. Data from [49].

The synthesis and evaluation of the cytotoxicity of novel pyridine Pt(II) complexes was described by Kutlu and coworkers [49] (Scheme 21). The complexes were cytotoxic against DLD-1 colon cancer cell lines and A549 human lung cancer cells. It should be noted that the [PtCl_2_L_2_] complex **55**, with the aminonitropyridine ligand, had a higher cytotoxic effect in comparison with the 3,4-dimethylpyridine ligand.

**Scheme 22:**
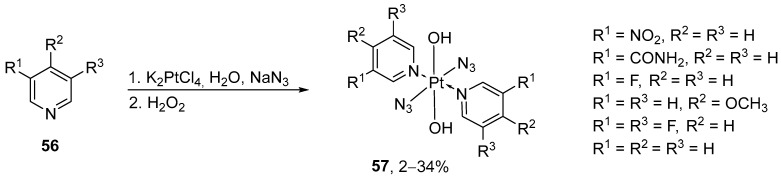
Synthesis of novel photoactive Pt(IV) complexes with heterocyclic ligands [50].

A series of novel photoactive Pt(IV) complexes with heterocyclic ligands was synthesized by Sadler and coworkers [50]. The synthetic route included the reaction of K_2_PtCl_4_ with pyridines **56** and sodium azide, followed by oxidation of the intermediate diazido complex with H_2_O_2_ (Scheme 22). The authors studied the photoactivation and phototoxicity of the resulting complexes **57** against bladder cancer cells. Nitropyridine complex (R^1^ = NO_2_) exhibited moderate non-light-induced cytotoxicity against the cell lines studied.

Nitrosyl iron complexes (NICs) were proven to be efficient inhibitors of enzymes in living organisms [51]. In particular, (N_2_H_5_)^+^[Fe(SR)_2_(NO)_2_]^−^ with R = 5-nitropyridin-2-yl was active against cyclic guanosine monophosphate phosphodiesterase (cGMP PDE) and sarcoplasmic reticulum (SR) Ca^2+^-ATPase. In addition, the NO-donating and hemolytic activity of similar iron complexes, with pyridine- and 5-nitropyridinethiolate ligands of the composition [Fe_2_(SR)_2_(NO)_4_], were studied [52]. The compound with the 5-nitropyridine ligand was much more active than the corresponding complex with unsubstituted 2-mercaptopyridine.

**Scheme 23:**
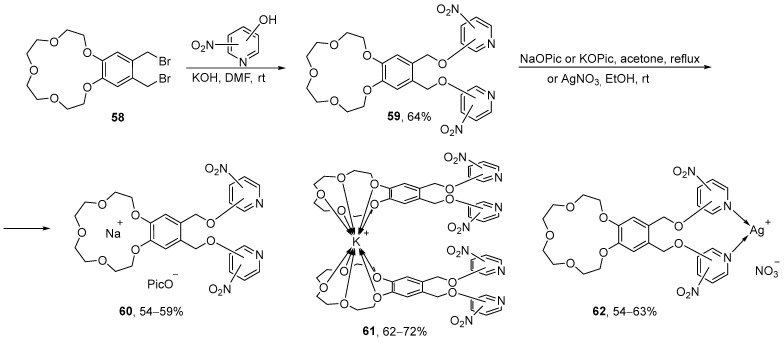
Nitropyridine complexes with antibacterial and antifungal activities. Data from [53].

Double-armed benzo 15-crown-5 compounds decorated with nitropyridine fragments were synthesized as ligands for the preparation of Na^+^, K^+^, and Ag^+^ complexes [53]. For this reason, 4′,5′-bis(bromomethyl)benzo-15-crown-5 **58** was treated with the corresponding hydroxynitropyridine under basic conditions (Scheme 23). The resulting ligands **59** formed metal complexes of different types, depending on the metal used. Thus, reaction with sodium picrate yielded 1:1 complex **60**, in which the crown fragment bound to Na^+^. In the case of potassium picrate, two cyclic ether parts coordinated to the potassium cation. Finally, reaction with silver nitrate produced complexes **62**, in which the pyridine nitrogen atoms coordinated to Ag^+^. All synthesized compounds exhibited moderate to good antibacterial (*E. coli*, *M. luteus*, *B. cereus*, *L. monocytogenes*, *S. Typhi*) and antifungal (*C. albicans*) activities at levels of commercial antibiotics. In particular, Ag^+^ complexes **62** can be used as antimicrobial agents against pathogenic microorganisms. It was determined that the activities of the synthesized compounds in Gram-negative bacteria were more effective than in Gram-positive bacteria.

## 4. Nitropyridines as Precursors and Intermediates of Bioactive Compounds

**Scheme 24:**
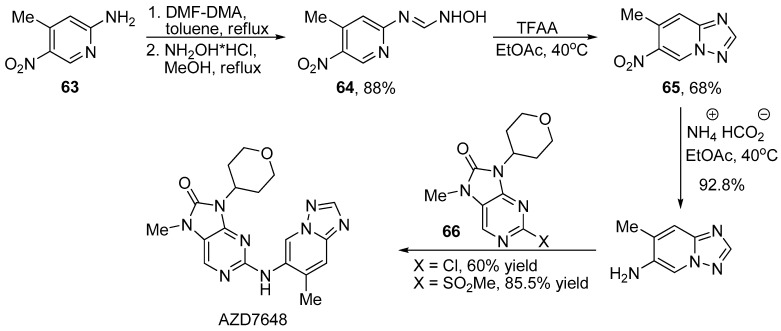
Synthesis of DNA-PK inhibitor AZD7648. Data from [54].

An efficient method for the synthesis of highly selective DNA-dependent protein kinase inhibitor AZD7648 was reported recently [54]. Commercially available 2-amino-4-methyl-5-nitropyridine **63** was used as the starting compound, which reacted with DMF DMA, followed by hydroxylamine (Scheme 24). The resulting hydroxyimine derivative **64** was cyclized to triazolo[1,5-*a*]pyridine **65**, and the nitro group was reduced with ammonium formate and Pd/C. Finally, Buchwald–Hartwig coupling with 2-chloropyrimidine derivative **66** produced the target AZD7648 in 60% yield. However, the authors achieved higher yields by replacing the chlorine atom of a pyrimidine intermediate with a methylsulfonyl group.

**Scheme 25:**
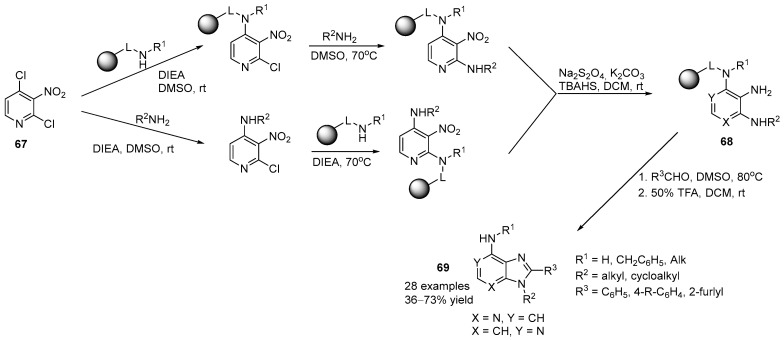
Solid-phase synthesis of PDE4 inhibitors **69**. Data from [55].

An efficient solid-phase synthesis of a library of substituted imidazo[4,5-*b*]pyridines with phosphodiesterase 4 and 7 inhibitory activity was described recently [55]. 2,4-Dichloro-3-nitropyridine **67** was used as a precursor, in which the chlorine atom in position 4 was more nucleofugal than Cl(2) and could be selectively substituted under the action of amines (free or immobilized to various types of resins) (Scheme 25). Next, the chlorine in position 2 was replaced by another amine, and the nitro group was converted into amine upon treatment with Na_2_S_2_O_4_ to yield 2,3,4-triaminopyridines **68**, the key intermediates in the synthesis of target imidazo[4,5-*b*]- and imidazo[4,5-*c*]pyridines **69**. As a result, the study revealed new potential anti-inflammatory agents for the treatment of autoimmune diseases. The most active compounds were found to inhibit PDE4 with an IC_50_ comparable to that of rolipram. They also exhibited a favorable pharmacokinetic profile, with a relatively long half-life in rats and good penetration to the brains of mice.

**Scheme 26:**
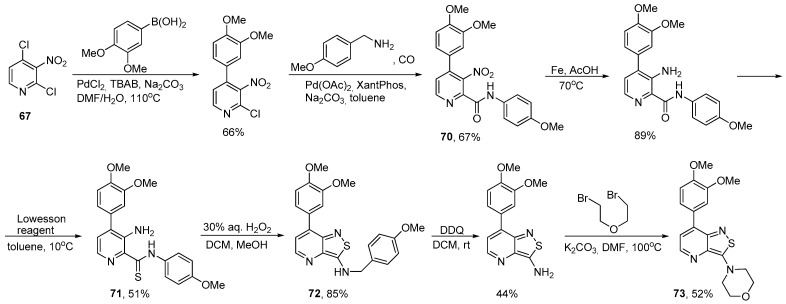
Approach to potential inhibitor of cyclin G-associated kinase [56].

A synthetic route to the previously unexplored 3,7-disubstituted isothiazolo[4,3-*b*]pyridine, a potential inhibitor of cyclin G-associated kinase, was elaborated starting from 2,4-dichloro-3-nitropyridine **67** [56]. Selective Suzuki–Miyaura cross-coupling at C(4), followed by Pd-catalyzed aminocarbonylation, produced compound **70**, which, after reduction of the nitro group and amide-thioamide interconversion, yielded intermediate **71** (Scheme 26). Next, the intramolecular cyclization of ortho-aminothioamide **71** to isothiazole **72** was carried out under the action of aqueous H_2_O_2_. Finally, debenzylation with DDQ and reaction with bis(2-bromoethyl)ether resulted in the formation of morpholine derivative **73**. The authors found that the target compound was inactive as a GAK inhibitor, unlike its 5- and 6-(3,4-dimethoxyphenyl) isomers.

**Scheme 27:**
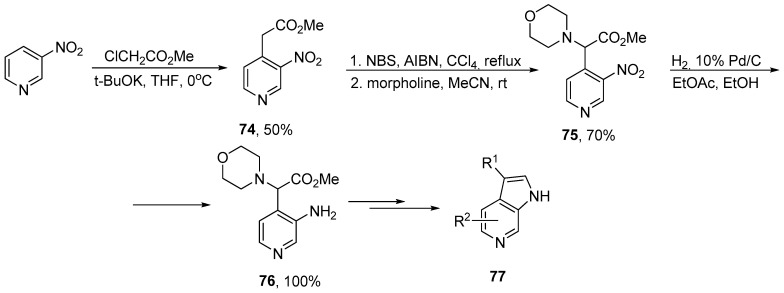
Synthesis of semicarbazide-sensitive amine oxidase (SSAO) inhibitors **77**. Data from [57].

Substituted 3-nitropyridines were used as precursors for the synthesis of semicarbazide-sensitive amine oxidase (SSAO) inhibitors [57]. An example of the synthetic routes is given in Scheme 27. Vicarious nucleophilic substitution of hydrogen with methyl chloroacetate under strong basic conditions provided compound **74**, which was then brominated and treated with morpholine to yield the corresponding morpholine derivative **75**. Reduction of the nitro group proceeded in the quantitative yield, resulting in the key intermediate **76** for the synthesis of target compounds **77**.

5-Nitropicolinic acid and 2-nitropyridine were reported to inhibit human NEU3—a key regulator of the beta1 integrin-recycling pathway, with IC_50_ concentrations of 40–79 nM [58].

**Scheme 28:**
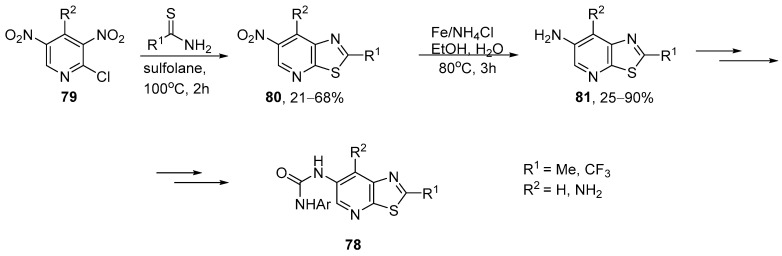
Synthesis of pyridine-based MALT1 inhibitors [59].

A series of functionalized thiazolo[5,4-*b*]pyridines **78** as potential MALT1 inhibitors in the treatment of autoimmune and inflammatory diseases and disorders were synthesized [59] (Scheme 28). In the first stage, the available 2-chloro-3,5-dinitropyridines **79** reacted with thioamides to yield 6-nitrothiazolo[5,4-*b*]pyridines **80**. The nitro group in **80** was reduced, leading to amines **81**, which then underwent a series of transformations to obtain target thiazolo[5,4-*b*]pyridines **78**. For all the synthesized compounds, the extent of inhibition of MALT1 protease activity was determined. The authors found that the MALT1 inhibition IC_50_ values of compounds **78** were in the 1–500 nM range.

**Scheme 29:**
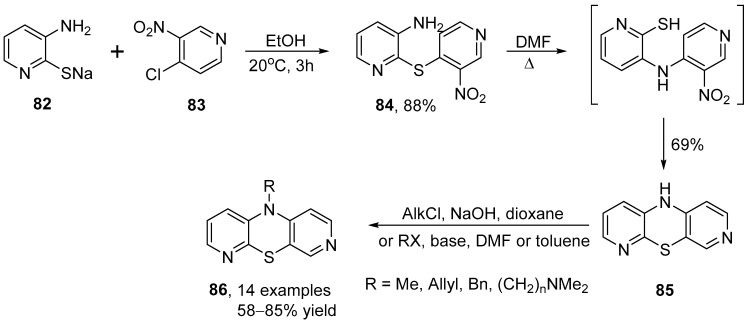
Synthesis of 10-substituted 3,6-diazaphenothiazines **86** based on nitropyridines. Data from [60].

A series of new 10-substituted 3,6-diazaphenothiazines were synthesized on the basis of nitropyridines and investigated for antitumor activity [60]. The reaction of sodium 3-amino-2-pyridinethiolate **82** and 4-chloro-3-nitropyridine **83** resulted in the formation of a sulfide through the nucleophilic substitution of a chlorine atom (Scheme 29). Heating this sulfide (compound **84**) in DMF produced 3,6-diazaphenothiazine **85**, presumably via a Smiles rearrangement. The target compounds **86** were obtained by the N-alkylation of compound **85** under the action of various alkyl- and hetaryl halides. 10*H*-3,6-diazaphenothiazines were found to be 10 times more active (IC_50_ < 0.72 mg/mL) than cisplatin against the glioblastoma SNB-19, melanoma C-32, and breast cancer MCF-7 cell lines. Diazaphenothiazines containing 2-pyrimidinyl and dimethylaminopropyl moieties exhibited selective activity against MCF-7 and C-32 cells. All 3,6-diazaphenothiazines are non-toxic or almost non-toxic against the normal human fibroblast (HFF-1) cell line in comparison with toxic cisplatin.

**Scheme 30:**
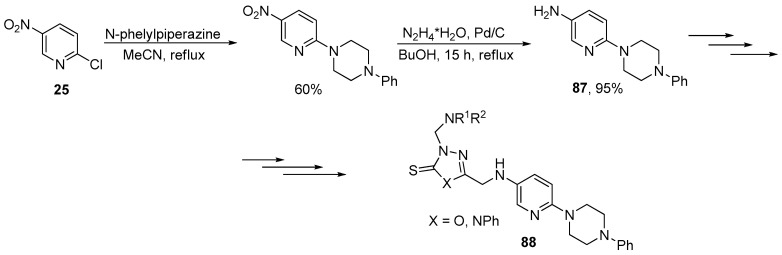
Synthesis of Mannich bases **88** active against prostate cancer cells. Data from [61].

6-(4-Phenylpiperazin-1-yl)pyridine-3-ylamine **87**, which is known to exhibit anticancer activity, was used as the leading compound for the design of new Mannich bases active against prostate cancer cell lines PC3, LNCaP, and DU145 [61]. Lead compound **87** was synthesized by the reaction of 2-chloro-5-nitropyridine **25** with N-phenylpiperazine, followed by the reduction of the nitro group (Scheme 30). The results revealed that the Mannich bases **88** exhibited moderate cytotoxic activity against cancer cells tested compared with the lead compound.

The same group reported on the synthesis of another series of anticancer compounds on the basis of 6-(4-phenylpiperazin-1-yl)pyridine-3-ylamine by incorporating biologically relevant thiourea, isothiocyanate, and thiazolidinone moieties [62].

**Scheme 31:**
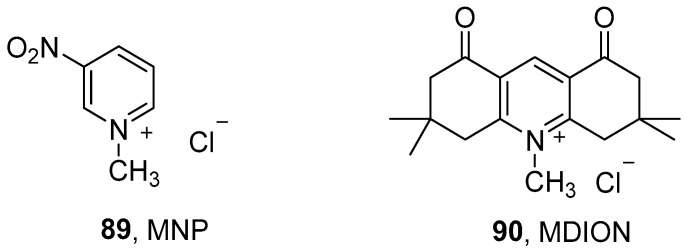
Antitumor N-methylpyridinium salts. Data from [63].

The antitumor effects of N-methyl pyridinium salts, namely 1-methyl-3-nitropyridine chloride (MNP, **89**) and 3,3,6,6,10-pentamethyl-3,4,6,7-tetrahydro-[1,8(2*H*,5*H*)-dion]acridine chloride (MDION, **90**), on sensitive leukemia HL60 cells and resistant topoisomerase II-defective HL60/MX2 cells were studied by Tarasiuk et al. [63] (Scheme 31). In the case of the HL60 cells, the IC_50_ values for MNP and MDION were 24.3 and 80.5 μM, respectively, while for the HL60/MX2 cells, they were 20.5 and 95.5 μM.

**Scheme 32:**
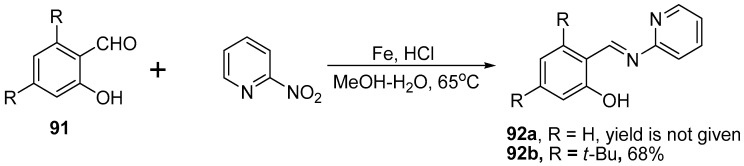
Synthesis of Shiff bases **92**. Data from [64].

An original and environmentally benign method for the synthesis of biologically active Shiff bases was reported in 2015 [64]. The reduction of 2-nitropyridine **91** with Fe/HCl in the presence of aromatic aldehydes provided azomethines **92** (Scheme 32). The authors performed antibacterial (*S. aureus*) and antifungal (*A. niger*) tests by the disc diffusion method; however, they did not provide certain MIC values.

**Scheme 33:**
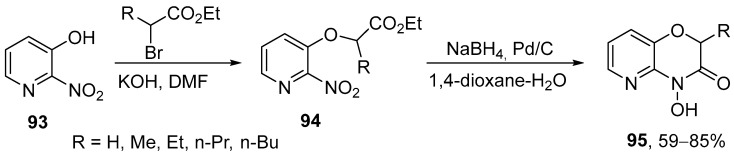
Synthesis of antimicrobial pyridoxazinone derivatives. Data from [65].

The synthesis and antimicrobial activity of pyridoxazinone series were reported recently [65]. O-alkylation of 3-hydroxy-2-nitropyridine **93** with 2-bromoalkanoic esters and subsequent reductive cyclization resulted in N-hydroxy-pyridoxazinone derivatives **95** in 59–85% yields (Scheme 33). One of the synthesized compounds (R = n-Bu) showed a MIC of 62.5 μg/mL against *C. albicans*, *C. glabrata*, and *C. tropicalis*. In addition, this compound showed a high antibacterial activity against *E. faecalis* (MIC 7.8 μg/mL) and *S. aureus* (MIC 31.2 μg/mL). Remarkably, the ethyl group in compound **95** (R = Et) increased the activity against *S. agalactiae* (MIC 62.5 μg/mL), which is higher than that of chloramphenicol.

**Scheme 34:**
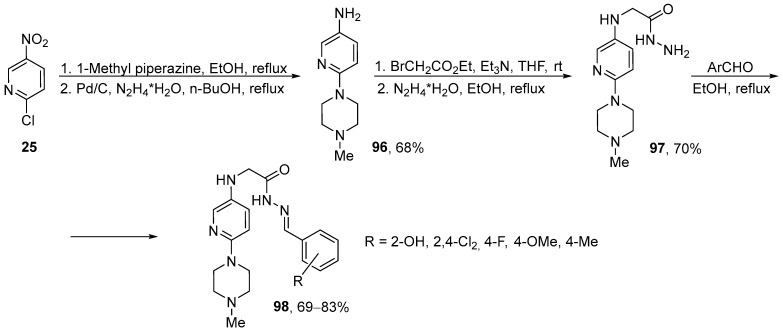
Synthesis of antimicrobial (pyridin-2-yl)piperazine derivatives **98**. Data from [66].

A series of new potential antimicrobial agents derived from (pyridin-2-yl)piperazine were synthesized by Boulebd’s group [66]. The reaction of 2-chloro-5-nitropyridine **25** with N-methylpiperazine and the subsequent reduction of the nitro group yielded amine **96**, which was then alkylated by ethyl bromoacetate and converted into the corresponding hydrazide **97** (Scheme 34). The latter was reacted with aromatic aldehydes to obtain hydrazones **98**. An antimicrobial activity study showed the resistance of *S. aureus* and *S. typhimurium* to all synthesized compounds. At the same time, a phenolic derivative (**98**, R = 2-OH) showed moderate antimicrobial activity against *B. subtilis* and *C. krusei*, with an MIC values of 62.5 μg/mL.

**Scheme 35:**
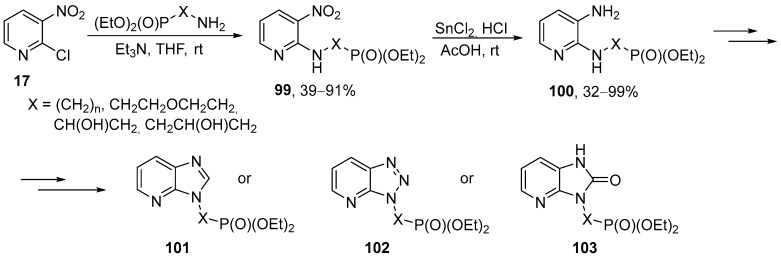
Synthesis of novel acyclic phosphonate nucleotide analogs **101**–**103**. Data from [67].

2-Chloro-3-nitropyridine **17** has been considered as a starting material in the synthesis of novel acyclic phosphonate nucleotide analogs incorporating azolopyridine fragments [67]. The synthetic scheme included the nucleophilic substitution of chlorine under the action of various ω-aminoalkylphosphonates and reduction of the nitro group (Scheme 35). Then, azole annulation was accomplished by reactions with HC(OEt)_3_, diazotization, or CDI, followed by hydrolysis of the phosphonic ester. All synthesized compounds **101**–**103** were tested for antiviral activity against DNA and RNA viruses and cytotoxicity. Some of them demonstrated activity against cytomegalovirus (EC_50_ = 76.47 μM; MCC > 100 μM) in human embryonic lung (HEL) cells and moderate activity against varicella zoster virus (VZV) (EC_50_ = 52.53–61.70 μM MCC > 100 μM) in HEL cells. No compounds were cytotoxic at a concentration of 100 μM.

**Scheme 36:**
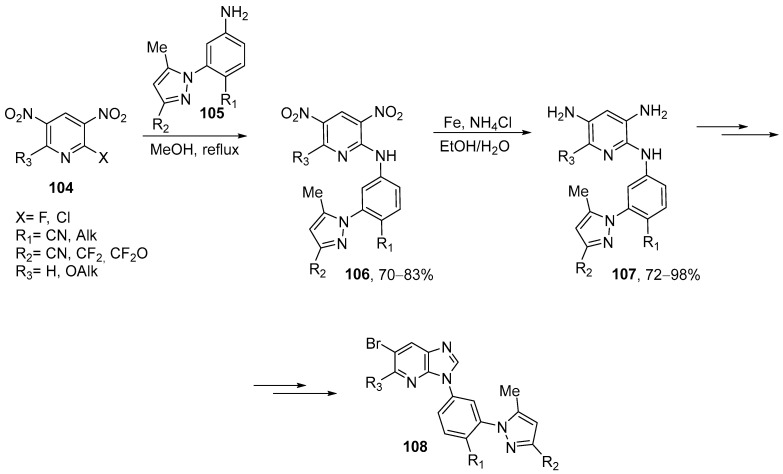
Synthesis of imidazo[4,5-*b*]pyridines **108** from 3,5-dinitropyridines. Data from [68].

Imidazo[4,5-*b*]pyridines, as the salt-inducible kinase (SIK) modulators for the treatment of rheumatoid arthritis, were described in patent [68]. The authors proposed available 3,5-dinitropyridine derivatives as the starting materials for the synthesis of desired compounds (Scheme 36). In the first stage, 2-fluoro- or 2-chloro-3,5-dintropyridines **104** reacted with the corresponding amine **105**, yielding compounds **106**. Then, the nitro groups were reduced, followed by condensation to yield diamines **107**, which were then converted into the target compounds **108**.

## 5. Nitropyridines in the Synthesis of Radiolabeled Compounds

Positron-emission tomography (PET) is used in clinical oncology, neurology, cardiology, and other fields for the imaging of tumors, the search for metastases, and for the clinical diagnosis of certain diffuse brain diseases, such as those causing various types of dementias. The administration of radiolabeled drugs is considered essential in order to keep track of the drug molecules throughout the body and excreta even after their transformation into different metabolites [69]. Today, PET mainly uses positron-emitting isotopes of elements from the second period of the periodic table: ^11^C, ^18^F, ^15^O, and ^13^N. Among them, ^18^F has optimal characteristics for use in PET, including the longest half-life and the lowest radiation energy. The relatively short half-life of ^18^F allows for obtaining high-contrast PET images with a low dose load on patients. The use of radiopharmaceuticals belonging to various classes of biologically active compounds makes PET a fairly universal tool in modern medicine. Therefore, the development of new radiopharmaceuticals and effective methods for synthesizing proven drugs is becoming a key stage in the development of the PET method [70].

**Scheme 37:**
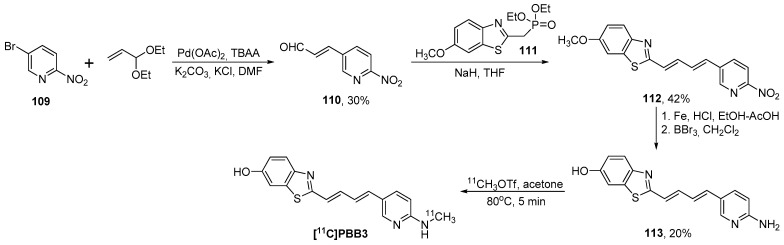
Synthetic route to PET tau tracer [^11^C]PBB3. Data from [71].

The synthesis of the PET tau tracer [^11^C]PBB3 compound for the imaging of Alzheimer’s disease was reported by Zheng and coworkers [71]. 5-Bromo-2-nitropyridine **109** was reacted with acroleine diethyl acetal under Heck reaction conditions, resulting in compound **110** in 30% yield (Scheme 37). Furthermore, the Wittig–Horner reaction with diethyl phosphonate **111** and reduction of the nitro group yielded amine **112**, which was then O-demethylated with BBr_3_. N-methylation with [^11^C]CH_3_OTf resulted in the target compound in 20–25% radiochemical yield.

**Scheme 38:**
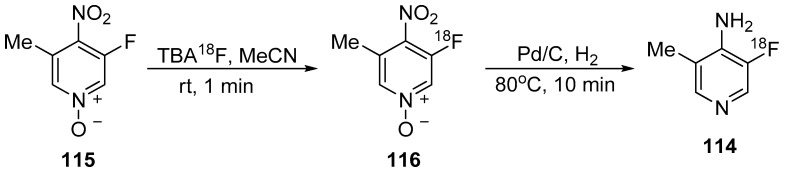
Radiochemical synthesis of [^18^F]4-amino-3-fluoro-5-methylpyridine. Data from [72].

Brugarolas and coworkers described the radiochemical synthesis of [^18^F]4-amino-3-fluoro-5-methylpyridine **114** based on 4-nitropyridine-N-oxide derivative **115** [72]. The authors used the ^18^F/^19^F isotope exchange method [73], followed by the Pd/C-mediated hydrogenation of both the nitro group and the N-oxide function (Scheme 38). The target compound was proven to effectively cross the blood–brain barrier in mice and was positioned as a promising candidate for neuroimaging applications, particularly for detecting demyelinated lesions in the brain.

**Scheme 39:**
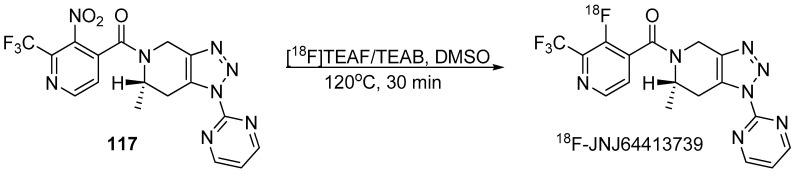
Radiosynthesis of P2X7R imaging agent ^18^F-JNJ64413739. Data from [74].

The automated radiosynthesis of P2X7R imaging agent ^18^F-JNJ64413739 was elaborated [74]. The key step involved nucleophilic aromatic substitution of 3-NO_2_ in substituted nitropyridine **117** under the action of a [^18^F]TEAF/TEAB mixture at 120 °C (Scheme 39). PET/CT imaging demonstrated higher radioactivity uptake in brain regions of the model rats with osteoporosis compared to the control group.

**Scheme 40:**
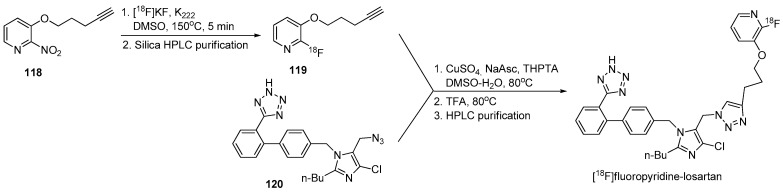
Synthesis of [^18^F]fluoropyridine-losartan. Data from [75].

A new approach to human PET imaging of the Angiotensin II type 1 receptor was elaborated recently [75]. The authors accomplished the synthesis of [^18^F]fluoropyridine-losartan with high molar activity and high chemical and radiochemical purity through the introduction of ^18^F by nucleophilic substitution of the nitro group with [^18^F]KF. The fluoropyridine and losartan fragments were combined together by azide–alkyne cycloaddition (Scheme 40).

**Scheme 41:**
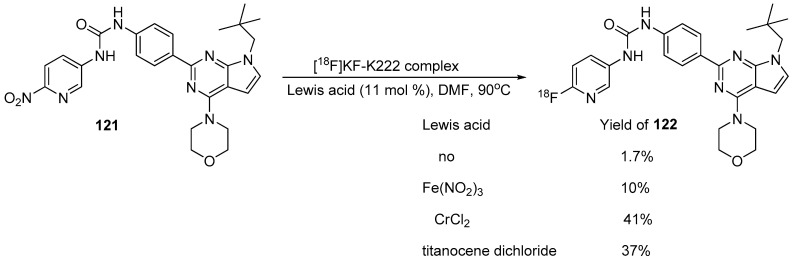
The synthesis of [^18^F]FMN3PU via replacement of the nitro group [76].

The influence of Lewis acid addition on the radiochemical yield of [^18^F]FMN3PU targeting leucine-rich repeat kinase 2 (LRRK2) was studied [76]. On addition of ferric nitrite, titanocene dichloride, or especially chromium(II) chloride, the radiochemical yield was increased to 41%, compared to 1.7% under conventional nucleophilic radiofluorination conditions (Scheme 41).

**Scheme 42:**
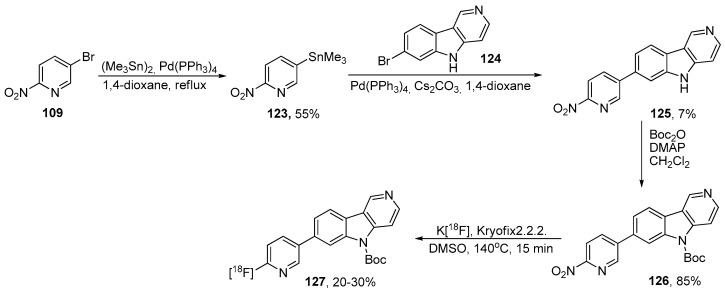
Synthesis of [^18^F]T807. Data from [77].

Finally, the synthesis of [^18^F]T807, a PET tau tracer for Alzheimer disease was reported by Zheng et al. [77] The authors proposed a reaction scheme starting with the Stille coupling of 5-bromo-2-nitropyridine **109** with 7-bromo-5*H*-pyrido[4,3-*b*]indole **124**, followed by Boc protection (Scheme 42). In the final step, nucleophilic substitution of the nitro group under the action of radiolabeled potassium fluoride occurred, accompanied by deprotection. This approach can be used for the synthesis of other ^18^F-radiotracers for PET imaging.

## 6. Conclusions

This analysis of publications from 2015 to 2024 shows that nitropyridines represent valuable scaffolds for the development of bioactive molecules and have garnered significant interest from researchers. Over the past decade, numerous practical applications of nitropyridines have emerged, such as their use as radiolabeled compounds for positron-emission tomography, ligands for coordination compounds showcasing diverse bioactivity, and more. Additionally, nitropyridines may serve as convenient and readily available precursors for a wide variety of promising complex bioactive molecules, including antitumor, antibacterial, and antifungal compounds, as well as herbicides and insecticides. From a chemist’s perspective, nitropyridines offer vast synthetic potential. The nitro group facilitates reactions with nucleophilic reagents and can be a source of other nitrogen functions, such as amino groups, azo linkages, hydroxylamines, etc. These functionalities pave the way to the development of fused heterocyclic systems. Furthermore, as demonstrated in this review, nitropyridines themselves exhibit useful biological activities.

Nitropyridines have been an underexplored class of compounds, likely due to the limited methods available for their functionalization. However, recent studies have introduced simple synthetic routes to polyfunctional derivatives of pyridine. We believe that this progress will encourage more active exploration of their biological properties. The number of publications focused on the chemistry of nitropyridines is steadily increasing, suggesting that this area will continue to develop in the future. Consequently, the field related to the creation of biologically active molecules based on nitropyridines is expected to remain in demand and relevant.

## Data Availability

Data sharing is not applicable.

## Abbreviations

The following abbreviations are used in this manuscript:

FDA	U.S. Food and Drug Administration
CNS	Central nervous system
IC_50_	Half-maximal inhibitory concentration
LD_50_	Median lethal dose
MIC	Minimum inhibitory concentration
MCC	Minimum cytotoxic concentration
PET	Positron emission tomography
DNA	Deoxyribonucleic acid
HIV	Human immunodeficiency virus
DDQ	2,3-Dichloro-5,6-dicyanobenzoquinone
CDI	1,1′-Carbonyldiimidazole
4-NPO	4-Nitropyridine-*N*-oxide
AgNPs	Silver nanoparticles
JAK2	Janus kinase 2
GSK3	Glycogen synthase kinase 3
SSAO	Semicarbazide-sensitive amine oxidase
cGMP PDE	Cyclic guanosine monophosphate-specific phosphodiesterase type 5
NEU3	Neuraminidase 3
MALT1	Mucosa-associated lymphoid tissue lymphoma translocation protein 1
VZV	Varicella zoster virus
DCC	N,N′-Dicyclohexylcarbodiimide
SIK	Salt-inducible kinase
